# Discrimination of benign from malignant breast lesions in dense breasts with model-based analysis of regions-of-interest using directional diffusion-weighted images

**DOI:** 10.1186/s12880-020-00458-3

**Published:** 2020-06-09

**Authors:** Alan I. Penn, Milica Medved, Vandana Dialani, Etta D. Pisano, Elodia B. Cole, David Brousseau, Gregory S. Karczmar, Guimin Gao, Barry D. Reich, Hiroyuki Abe

**Affiliations:** 1 Alan Penn & Assoc., Inc., 14 Clemson Ct, Rockville, MD 20810 USA; 2 Department of Radiology, The University of Chicago, 5841 S. Maryland Ave. MC 2026, Chicago, IL 60637 USA; 3 Beth Israel Deaconess Medical Center, 330 Brookline Ave, Boston, MA 02215 USA; 4 American College of Radiology, Two Liberty Place, Philadelphia, PA 19102 USA; 5 Providence Cedars-Sinai Tarzana Medical Center, 18321 Clark Street, Tarzana, CA 91356 USA; 6 Department of Public Health Sciences, The University of Chicago, 5841 S. Maryland Ave. MC 2000, Chicago, IL 60637 USA

## Abstract

**Background:**

There is an increasing interest in non-contrast-enhanced magnetic resonance imaging (MRI) for detecting and evaluating breast lesions. We present a methodology utilizing lesion core and periphery region of interest (ROI) features derived from directional diffusion-weighted imaging (DWI) data to evaluate performance in discriminating benign from malignant lesions in dense breasts.

**Methods:**

We accrued 55 dense-breast cases with 69 lesions (31 benign; 38 cancer) at a single institution in a prospective study; cases with ROIs exceeding 7.50 cm^2^ were excluded, resulting in analysis of 50 cases with 63 lesions (29 benign, 34 cancers). Spin-echo echo-planar imaging DWI was acquired at 1.5 T and 3 T. Data from three diffusion encoding gradient directions were exported and processed independently. Lesion ROIs were hand-drawn on DWI images by two radiologists. A region growing algorithm generated 3D lesion models on augmented apparent-diffusion coefficient (ADC) maps and defined lesion core and lesion periphery sub-ROIs. A lesion-core and a lesion-periphery feature were defined and combined into an overall classifier whose performance was compared to that of mean ADC using receiver operating characteristic (ROC) analysis. Inter-observer variability in ROI definition was measured using Dice Similarity Coefficient (DSC).

**Results:**

The region-growing algorithm for 3D lesion model generation improved inter-observer variability over hand drawn ROIs (DSC: 0.66 vs 0.56 (*p* < 0.001) with substantial agreement (DSC > 0.8) in 46% vs 13% of cases, respectively (p < 0.001)). The overall classifier improved discrimination over mean ADC, (ROC- area under the curve (AUC): 0.85 vs 0.75 and 0.83 vs 0.74 respectively for the two readers).

**Conclusions:**

A classifier generated from directional DWI information using lesion core and lesion periphery information separately can improve lesion discrimination in dense breasts over mean ADC and should be considered for inclusion in computer-aided diagnosis algorithms. Our model-based ROIs could facilitate standardization of breast MRI computer-aided diagnostics (CADx).

## Background

There is an increasing interest in the use of non-enhanced breast diffusion-weighted imaging (DWI). This is due in part to concern of harm from gadolinium, the enhancing agent used with dynamic-contrast-enhanced MRI (DCEMRI) [1, 2]. A common lesion classifier is the mean value of apparent-diffusion coefficient (ADC) calculated over a lesion region-of-interest (ROI). However, lesion ROIs can include cysts and/or areas of necrosis, or suffer from volume-averaging at boundaries – all of which can degrade the effectiveness of mean ADC as a discriminator. Several studies have investigated methods of extracting and evaluating only the relevant portions, or sub-ROIs, of the original full-lesion ROI. They have concluded that ROI placement significantly influences reported ADC values in breast tumors and that smaller ROIs are frequently associated with improved discrimination [3–5].

Methods of defining sub-ROIs have varied widely among investigators. For example, including manually avoiding areas identified on pre-contrast T1- or T2-weighted images [6, 7], using very small (3–4 pixel) subregions with low intensity signals [4], and covering the full-lesion ROI with circular sub-ROIs of fixed size and selecting the sub-ROI with lowest ADC value [8] have been tested. Differences in sub-ROI selection methodologies and imaging protocols have led to reported results that are hard to compare across studies [9, 10]. The method of defining the ROI affects lesion-averaged ADC values, since drawing an ROI by hand or placing geometric shapes over or within the lesion can introduce bias by including background pixels and/or excluding lesion pixels at the boundary [8, 11–14]. Previous studies showed that ADC values for malignant tumors depend upon how much peri-tumor tissue is included in the evaluation. Specifically, ADC of the central part of a malignant lesion was significantly lower than ADC of the whole lesion, while there was no significant difference for benign lesions [7, 15]. Partridge et al. and Zeilinger et al. noted limitations of hand-drawn ROIs, including ROI reproducibility and accuracy, and the difficulty in propagating ROIs from DCEMRI to DWI images [14, 16].

We present a model-based approach that introduces three methodological novelties for DWI data acquisition and post-processing. First, we obtain and utilize separately the information from the three diffusion-encoding gradient datasets. Second, we introduce a region-growing algorithm for generating 3D, topologically connected lesion volume-of-interest (VOI) models from which 2D ROIs are derived. Finally, rather than analyzing the lesion as a single ROI, we define lesion-core and periphery sub-ROI (peri-lesion) and derive separate features for the two. This was motivated by earlier studies showing that analysis of peri-lesion tissue can provide a discriminatory feature (morphological blooming) in DCEMRI [17] and in ADC analysis [15].

The purpose of this work is to present a new approach that introduces the three methodological improvements and to demonstrate that it leads to enhanced diagnostic performance over using the mean ADC of the lesion ROI as a classifier of malignant vs benign lesions. Only patients with dense breasts are included in the study, as this is the population with the worst diagnostic performance on mammography and thus with the greatest need for improved imaging techniques.

## Methods

### Patient recruitment and imaging protocol

The study was performed under an IRB-approved protocol, with informed consent obtained from all subjects. Patients with breast lesions found on mammographic and/or sonographic exams were recruited prospectively before breast biopsy was performed. Subjects who had undergone prior treatment were not accrued, as that could distort diffusion signals. Fifty-five patients with 69 lesions (38 malignant; 31 benign) were imaged between Jan. 1, 2015 and Nov 15, 2016 using 1.5 T and 3.0 T MR systems. Lesions, as annotated by Radiologist 1 who had access to all imaging and clinical data, were categorized by maximum in-plane area as follows: small-medium (55 lesions: 0.14 cm^2^–6.74 cm^2^) and large (6 lesions: 8.63 cm^2^–49.30 cm^2^).

The data set contained no lesions with sizes between 6.74 cm^2^ and 8.63 cm^2^, and cases with ROIs larger than that gap were excluded. The diameter of tumors having sizes within the range of the gap is approximately 3 cm. Clinical studies suggest that there is a low risk of local recurrence with breast conservation surgery in invasive breast cancers that are less than 3 cm in diameter [18]. Thus, choosing cancers less than 3 cm is clinically meaningful and minimized the size bias of discriminating features. The threshold 7.50 cm^2^ was nominally used to represent the gap 6.74 cm^2^ to 8.63 cm^2^.

No lesions were excluded because of imaging problems or patient motion. The study set included 49 masses (26 cancer; 23 benign). 12 non-mass enhancements (NME) (8 cancer; 4 benign) and two benign lesions that were identified on mammography as calcifications 1 and architectural distortion 1 but negative on DCEMRI. Twelve lesions were found in extremely dense breasts and 51 in heterogeneously dense breasts. All lesions underwent image-guided biopsies following MR imaging.

The subjects underwent DWI, non-fat suppressed T2-weighted imaging, and DCEMRI using dedicated 16-channel Mammotrack phased array breast coils (Philips Healthcare, Best, Netherlands), at a 1.5 T Achieva (Philips Healthcare, Best, Netherlands; 1 benign; 3 malignant lesions) and a 3 T Achieva (Philips Healthcare, Best, Netherlands; 30 benign; 35 malignant lesions). Diffusion weighted images were acquired prior to the administration of gadolinium-based contrast agent and the acquisition of DCEMRI. Spin-echo echo-planar imaging (SE-EPI) was used to generate diffusion weighted images and corresponding ADC maps in the axial plane, with imaging parameters shown in Table 1. DWI data were acquired, retained, and analyzed individually for each of the three diffusion gradient encoding directions.

**Table 1:** Imaging parameters for the SE-EPI DWI sequence

	Philips Achieva 1.5 T	Philips Achieva 3.0 T
TR [ms]	16,860–16,960	10,546–13,863
TE [ms]	80.1	63.9–67.5
Field-of-view [mm^2^]	300 × 300–330 × 330	300 × 300–390 × 390
In-plane resolution [mm]	1.15–1.25	1.04–1.25
Slice thickness [mm]	2.5	2.5
Number of slices	80	65–80
b values [s/mm^2^]	0, 800	0, 800

### Image analysis

**Fig. 1:**
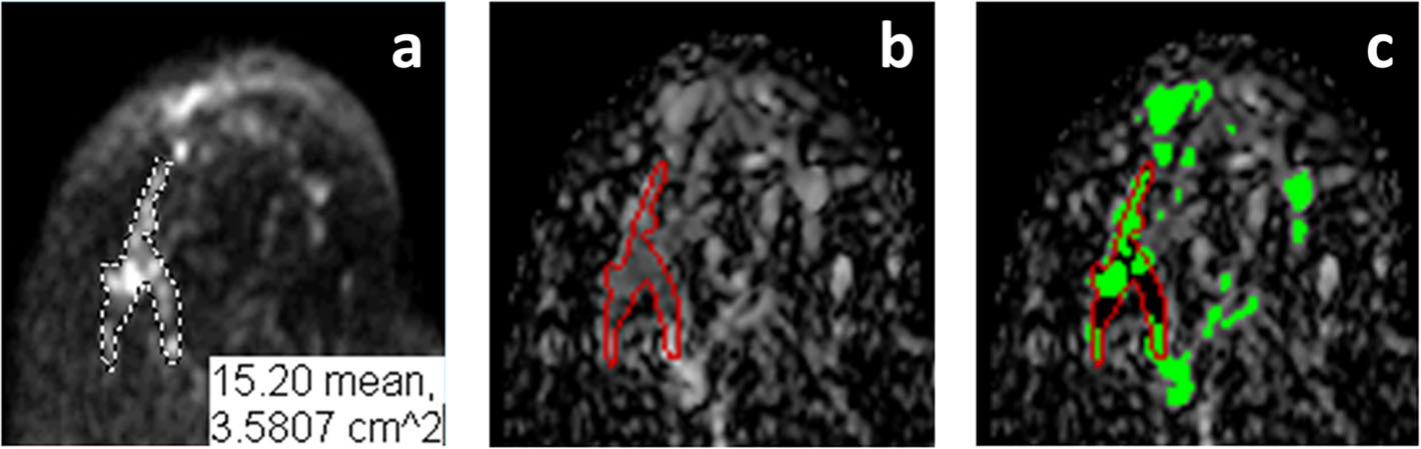
2D cross-section of the model 3D lesion VOI

Images were analyzed by two fellowship-trained breast radiologists with over 10 years of experience (HA, VD) who read MRI as part of their clinical practice. Radiologist 1, who was familiar with clinical results and had access to mammographic, sonographic and DCEMRI images in addition to DWI images, selected one of the ADC, b = 0 s/mm^2^, b = 50 s/mm^2^ or b = 800 s/mm^2^ series for lesion delineation based on the reader’s assessment of lesion visibility. Radiologist 1 then selected the set of axial slices containing the lesion that would be annotated and drew lesion ROIs on each of the selected axial slices. The slice with the largest ROI was designated the “index slice.” Annotations of Radiologist 1 were drawn on interpolated images (512 × 534–1274 × 994 pixels) using the 1680 × 1050 HP Compaq LA2205wg monitor (Hewlett-Packard, Palo Alto, CA) which recorded, for each annotated slice, the size of the marked ROI in cm^2^ (see, e.g., Fig. 1a). The annotated images were then down-sampled to the original size of the DICOM images (240 × 240–336 × 336 pixels). Pixels on the down-sampled images have signal contribution from multiple interpolated pixels and were included in the final ROI when a given percentage of contributing pixels had been included in the hand-drawn ROI. This percentage was selected such that the final ROI size in cm^2^ was the closest to that on the interpolated image, for each case.

To simulate reading conditions of a non-contrast MRI exam, Radiologist 2 was presented with mammographic, sonographic, and DWI images but was blinded to clinical report results and DCEMRI images. Radiologist 2 was also presented with arrows pointing to the approximate location of the lesion on b = 0 and 800 s/mm^2^ images and with ADC maps of the index slice. Radiologist 2 used these images to assess the location and extent of the lesion, selected the index DW image to be annotated independently from Reader 1 selection, and hand drew the lesion ROI on this index slice using Mi-Forms Designer v.11 (Mi-Corporation, Durham, NC) on a Lenovo Thinkpad X230 Tablet PC. Radiologist 2 annotated images interpolated to a fixed-size template of 340 × 340 pixels and the annotated images were then down-sampled to the original size of the DICOM images. Pixels in the down-sampled images were included in the ROI if at least 50% of contributing pixels were in the ROI on the 340 × 340 image.

Directional ADC maps were constructed for each diffusion gradient encoding dataset (Eq. 1):

1 $$ {\mathrm{ADC}}_{\mathrm{d}}=\left(1/{\mathrm{b}}_{800}\right)\ast \ln\ \left({\mathrm{S}}_0/{\mathrm{S}}_{\mathrm{d},800}\right) $$

where S_0_ is the signal intensity in DW image obtained at b = 0 s/mm^2^ and S_d,800_ is the signal intensity in DW image obtained for the given diffusion encoding gradient direction (d = r, p, or s for readout, phase encoding, and slice encoding direction, respectively) and b = 800 s/mm^2^. An augmented directional ADC map (auADC_d_) was constructed for each of the three directional ADC_d_ maps by multiplying the pixel intensity on the directional ADC_d_ map by the corresponding pixel intensity of the S_0_ image (Eq. 2).

2 $$ {\mathrm{auADC}}_{\mathrm{d}}={\mathrm{S}}_0\ast {\mathrm{ADC}}_{\mathrm{d}}={\mathrm{S}}_0\ast \left(1/{\mathrm{b}}_{800}\right)\ast \ln\ \left({\mathrm{S}}_0/{\mathrm{S}}_{\mathrm{d},800}\right) $$

S_0_ is a non-directional, T2-weighted image with increased lesion conspicuity that is low-resolution but with spatial distortion that matches that of higher b-value DW images and ADC maps. Using augmented auADC_d_ maps was previously found to perform well for lesion ROI definition [19].

From ROIs drawn by Radiologists 1 and 2, independently, and for each direction separately, 3D VOI models were computer-generated around the lesion by including spatially connected voxels with auADC_d_ signal higher than a certain threshold. The 3D model VOIs cross-section in the index slice generally differed from the 2D hand-drawn ROI, and the threshold was selected so that the Jaccard similarity index between these was maximized [20]. The overlap of the hand-drawn ROI and the 3D VOI cross-section in the index slice defined the “core-lesion sub-ROI”, while the remainder of the 2D hand-drawn ROI constituted the “peri-lesion sub-ROI”. Thus, three direction-dependent sets of core- and peri-lesion sub-ROIs were defined for each reader, and quantitative differences between these directional sub-ROIs were exploited for enhanced diagnostic purposes.

Figure 1 illustrates the index slice containing an invasive lobular carcinoma (ILC) in a 57-year-old patient with heterogeneously dense breasts imaged at 3.0 T. Figure 1a shows the original hand-drawn ROI from Radiologist 1 on a b = 800 s/mm^2^ image. Figure 1b shows the hand-drawn ROI superimposed in red on the ADC map. Figure 1c shows green pixels that represent the cross-section with the 3D computer lesion model constructed from auADC_r_ values. The lesion model in Fig. 1c is topologically connected in 3D, but not in 2D, as shown. Green pixels that lie within the hand-drawn ROI form the lesion-core; black pixels that are within the hand-drawn ROI but are not in the computer model form the peri-lesion.

### ROC analysis

The ROC analysis was targeted for a binary classification, where positive samples were malignant lesions and negative samples were benign lesions. Sensitivity was defined as the ratio of the number of true positives over the sum of the numbers of true positives and false negatives. Specificity was defined as the ratio of the number of true negatives over the sum of the numbers of true negatives and false positives. Sensitivity was evaluated at specificity equal to 90% on the ROC curves [21]. Within core- and peri-lesion sub-ROIs, a threshold of ADC = 1.37 mm^2^/s was selected to separate “benign-like” (ADC ≥ 1.37 mm^2^/s) from “cancer-like” voxels (ADC < 1.37 mm^2^/s), based on a previously published study [19]. The quantitative feature defined for lesion-core ROIs was the standard deviation of area covered by cancer-like voxels (SDAC) over the three directions. Thus, SDAC directly quantifies differences between directional DWI datasets, and higher SDAC values indicate higher probability of cancer. If the mean area covered by cancer-like pixels was < 6.67 mm^2^, (i.e., total area for three directions < 20 mm^2^) the cancer-like pixels were assumed to be spurious and SDAC was forced to 0, indicating benignity. The quantitative feature defined for peri-lesion ROIs was the area covered by cancer-like pixels minus the area covered by benign-like pixels (ACMB) over all three directions, with negative values permitted. Higher ACMB values indicated higher probability of cancer. SDAC was combined with ACMB into a single ROC model-based classifier (MBC), using logistic regression with 5-fold cross validation. The baseline discrimination classifier for each reader was the mean ADC value over the hand-drawn ROIs. Mass lesions and lesions smaller than 1 cm^2^ were also independently analyzed.

### Statistical analysis

Differences between benign and malignant ROI sizes were evaluated using a 2-sided t-test, with significance level 0.05. Diagnostic performance of mean ADC, SDAC, ACMB, and MBC was evaluated using pROC and cvAUC packages in R (http://cran.us.r-project.org) [21]. Logistic regression with repeated stratified 5-fold cross-validation was used. Binormal ROC curves were constructed using cross-validated model parameters..

The Dice Similarity Coefficient (DCS) was used to evaluate inter-observer variability between ROIs resulting from Radiologist 1 and Radiologist 2 readings. The DCS values for each lesion averaged over the three directional algorithm-generated lesion-core sub-ROIs were compared to the DSC values for hand-drawn lesion ROIs, using the Wilcoxon signed-rank test. The McNemar test was used to compare percentages of cases with DSC greater than 0.8 (indicating substantial agreement) [22, 23].

## Results

**Fig. 2:**
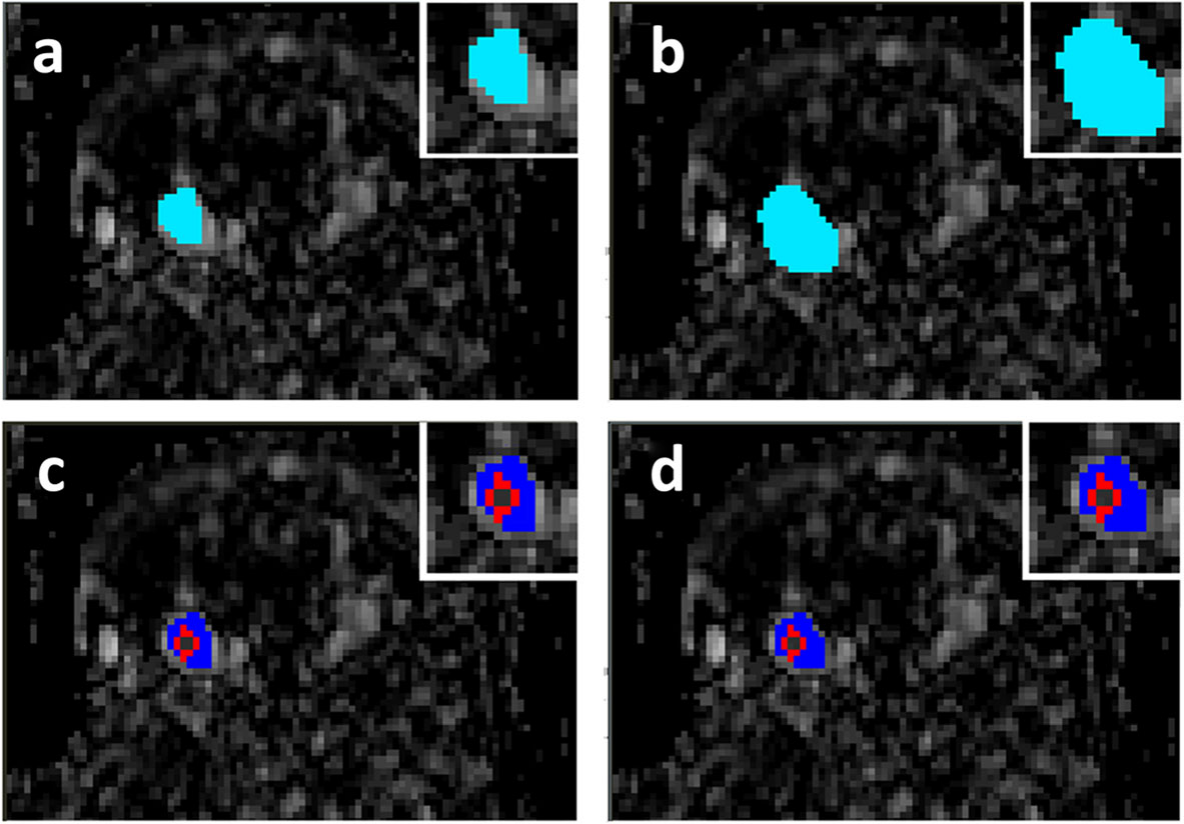
Definition of lesion core and lesion periphery sub-ROIs

Figure 2 shows the hand-drawn lesion ROIs (light blue, a and b) and lesion-core pixel sets (c and d) obtained from phase directional ADC_p_ map as defined by Radiologist 1 (left) and Radiologist 2 (right), in a 63-year-old patient with heterogeneously dense breasts with an invasive ductal carcinoma (IDC) lesion. The red and blue pixels in Fig. 2c-d mark the lesion core with red indicating cancer-like (ADC < 1.37) and blue indicating benign-like (ADC ≥ 1.37) voxels.

The mean sizes and standard deviations of study lesions, as recorded from annotations by Radiologist 1, were 154 ± 165 mm^2^ (min, median, max: 16, 80, 673 mm^2^) and 179 ± 142 mm^2^ (min, median, max: 14, 121, 463 mm^2^) for benign and malignant lesions, respectively (*p* = 0.52). The mean DSC values over study cases were 0.56 for hand-drawn ROIs (0.57 and 0.55 in cancer and benign cases, respectively) vs 0.66 for computer-generated lesion-core sub-ROIs (0.63 and 0.70 in cancers and benign cases, respectively) (*p* < 0.001). For hand-drawn ROIs, 12.7% of cases had mean DSC > 0.8 (11.8 and 13.8% in cancer and benign cases, respectively), vs 46.0% in algorithm-generated lesion core sub-ROIs (38.2 and 55.2% in cancer and benign cases, respectively). This difference was statistically significant (p < 0.001).

**Fig. 3:**
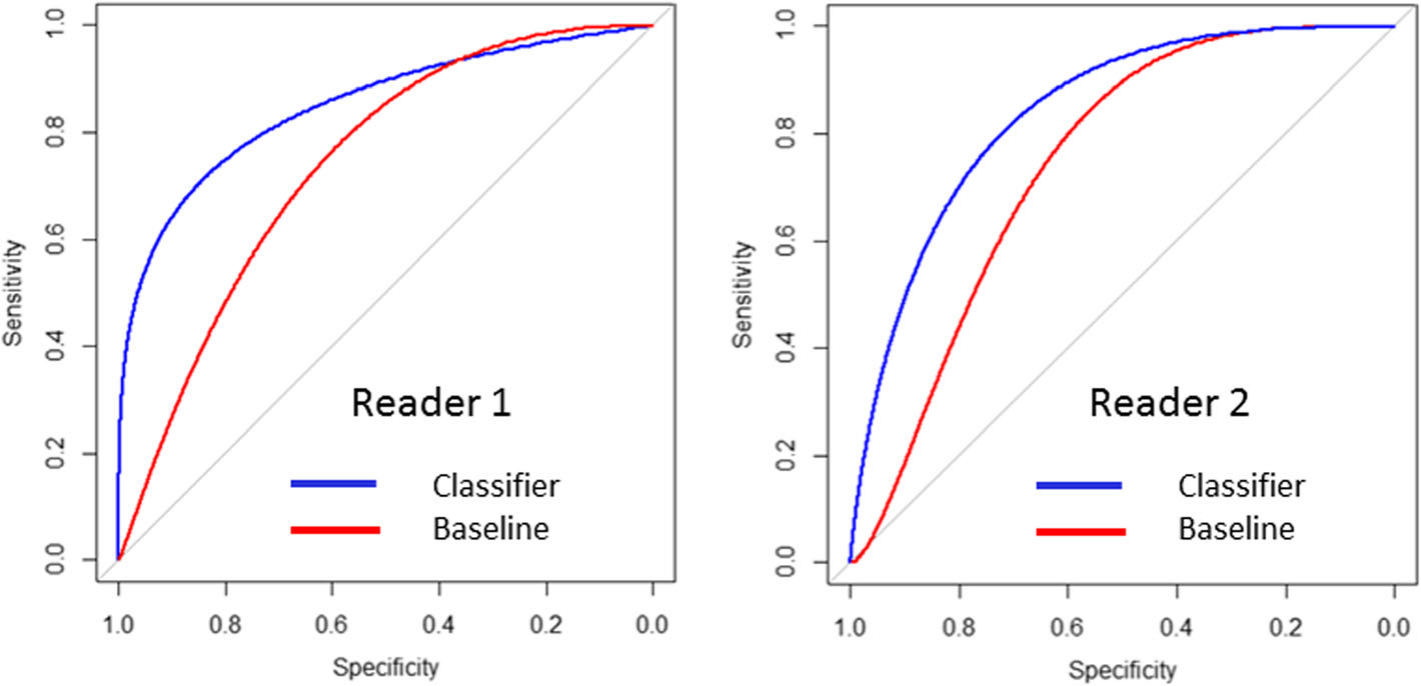
Binormal ROC curves for baseline and model-based classifier for the two readers

Figure 3 shows the corresponding baseline (mean ADC) and overall model-based classifier binormal ROC curves for Radiologist 1 and Radiologist 2. At specificity of 90% (3 FP; 26 TN), sensitivity for Radiologist 1 improved from 27 to 64% and sensitivity for Radiologist 2 improved from 19 to 49% for model-based classifier over baseline.

Table 2 shows ROC AUC values for baseline discrimination, ACMB, SDAC, and the overall model-based classifier for the two radiologists. Table 3 shows ROC AUC values for baseline discrimination, ACMB, and SDAC for small lesions (areas < 1 cm^2^; *n* = 29) as marked by Radiologist 1. Excluding from analysis the 4 lesions obtained at 1.5 T field strength did not materially change the AUC values. For Radiologist 1: AUCs for 3.0 T cases only were 0.755 and 0.860, for mean ADC and MBC, respectively, compared to AUC values for all cases of 0.748 and 0.850. For Radiologist 2: 3.0 T cases had AUC values of 0.740 and 0.843 compared to values for all cases of 0.743 and 0.829.

**Table 2:** ROC analysis results

Radiologist	Feature (No cases)	AUC
Radiologist 1	baseline (63)	0.748
	ACMB (63)	0.830
	SDAC (63)	0.846
	MBC (63)	0.850
	Masses (49): baseline / MBC	0.789/0.930
Radiologist 2	baseline (63)	0.743
	ACMB	0.831
	SDAC	0.775
	MBC (63)	0.829
	Masses (49): baseline / MBC	0.760/0.874

baseline = mean ADC of hand-drawn ROI.

MBC = model-based classier

**Table 3:** ROC analysis results for lesions smaller than 1 cm^2^

Radiologist	Feature (29 cases)	AUC
Radiologist 1	baseline	0.670
	ACMB	0.694
	SDAC	0.782
	MBC	0.747
Radiologist 2	baseline	0.707
	ACMB	0.760
	SDAC	0.667
	MBC	0.726

baseline = mean ADC of hand-drawn ROI.

**Fig. 4:**
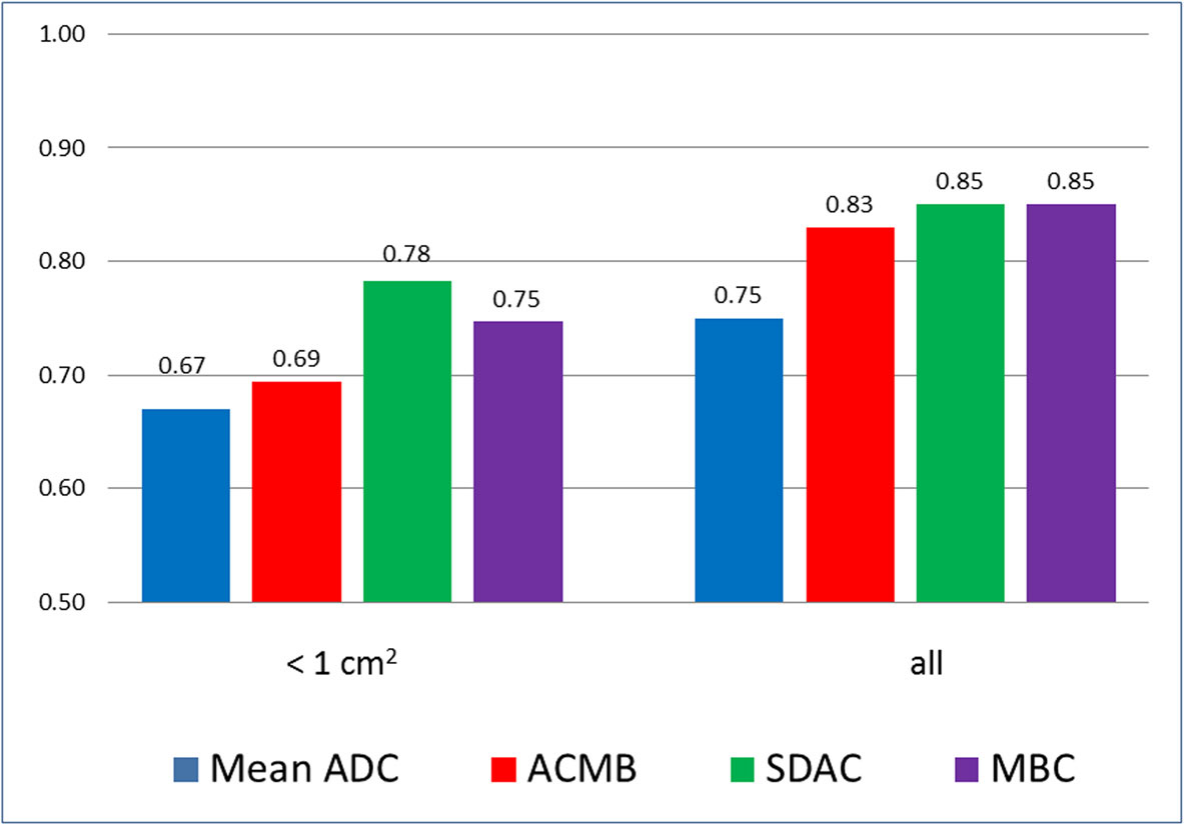
AUC values for baseline, ACMB, SDAC, and MBC, by lesion size

Figure 4 shows the AUC values computed for lesions as marked by Radiologist 1 for lesions with areas less than 1 cm^2^ (n = 29) and for all lesions (*n* = 63).

## Discussion

We have tested the performance of a model-based classifier for characterizing breast lesions, introducing three new techniques. First, we used a region-growing algorithm for generating topologically connected 3D lesion VOI models from which 2D lesion ROIs are derived. This algorithm operates on augmented directional ADC maps, where values of the directional ADC map are multiplied with corresponding values of the b = 0 s/mm^2^ DW image. Second, we retained and utilized the information from the three diffusion-encoding direction datasets separately, thus defining three different model-based sets of 3D VOIs and 2D ROIs. Finally, we divided the lesion ROI into lesion-core and peri-lesion sub-ROIs and derived distinct features for each which are combined into the overall model-based classifier. The improvements over baseline ROC AUC values validate our approach.

Our results indicate that inter-observer variability could be improved if lesion-core ROIs are standardized by employing algorithm-based definition. Improved inter-observer variability is important for both the clinical application of breast lesion discrimination and for development of CADx systems for breast cancer detection and diagnostics. We observed an increase in the percentage of cases with substantial agreement for lesion-core sub-ROIs. This demonstrates the benefit of computer-based ROI selection and potential for standardization of ADC measurement between observers and, potentially, institutions and manufacturers.

For both radiologists, our model-based classifier resulted in improvement in AUC over that obtained from mean ADC. Improvement in AUC on small lesions was found for both radiologists using self-training on their respective data sets; these results suggest that it may be possible to improve discrimination of small lesions using the methods introduced here, but a study with a larger set of small lesions is required to validate that hypothesis. For lesions with areas less than 1 cm^2^ in the index slice, the mean ADC performed especially poorly and for Radiologist 1, SDAC – the lesion-core feature derived from differences in directional DW images – was responsible for most of the improvement in AUC. Using only three gradient directions, rather than a more time-intensive DTI sequence, preferentially labels as anisotropic the lesions that have a suitable orientation relative to the directions of the gradients. However, our results demonstrate that even the limited anisotropy information derived from DWI is helpful in improving diagnostic accuracy. This is most strikingly true for small lesions, where the discrimination task is especially challenging. As the imaging time is not increased, this is a penalty-free method for improving diagnostic performance.

Radiologist 1 defined hand-drawn lesion ROIs with access to all the clinical and radiological information, which is the typical scenario for evaluation of suspicious findings. Radiologist 2 drew lesion ROIs without access to DCEMRI information. Importantly, MR imaging was performed prior to lesion biopsy. Thus, our results are relevant to and could help improve accuracy of either DCEMRI-based diagnostic or screening MRI exams (Radiologist 1 analysis), or of non-contrast-enhanced screening exams (Radiologist 2 analysis).

There are several limitations to this study. First, the two Radiologists drew ROIs with different prior information, which could introduce variability. However, even with these differences we have observed improved inter-reader agreement in lesion core ROIs. This indicates that our method is more robust to variations in methodology than using simple hand-drawn ROIs. Second, while this prospective study was designed to include and analyze all non-excluded cases, most of the cases (59/63) were imaged on 3.0 T systems. In our sample however, restricting the analysis to cases imaged at 3.0 T resulted in minimal differences in AUC values without materially impacting the results. Third, we used a fixed ADC cutoff for SDAC and ACMB definitions, based on earlier results, and it is possible that a more optimal cutoff value would yield improved discrimination. Finally, this study was restricted to women with dense breasts and it remains to be seen how these results translate to the general population.

## Conclusions

The combination of (a) independent evaluation of DWI directional signals, (b) model-based VOIs, and (c) features derived separately from lesion-core and peri-lesion ROIs defined on augmented ADC maps could improve discrimination of benign from malignant breast lesions in women with dense breasts. Inter-observer variability among readers could be reduced by modeling lesions as 3D, topologically connected VOIs from which 2D ROIs are derived. The use of limited anisotropic information derived from directional DWI datasets improves diagnostic accuracy without an acquisition time penalty. The methods introduced here could potentially increase the diagnostic accuracy of both DCEMRI-based and non-contrast screening breast MRI exams.

## Data Availability

Availability of human subject data is subject to restrictions under the IRB. Datasets used for statistical analysis in the current study are available from the corresponding author on reasonable request.

## Abbreviations

AUC: Area under the curve
ACMB: Area cancer- minus benign-like voxels
ADC: Apparent-diffusion coefficient
auADC: Augmented ADC
CADx: Computer-aided diagnostics
DCEMRI: Dynamic contrast-enhanced MRI
DICOM: Digital imaging and communications in medicine
DSC: Dice similarity coefficient
DTI: Diffusion tensor imaging
DWI: Diffusion-weighted imaging
IDC: Invasive ductal carcinoma
ILC: Invasive lobular carcinoma
MBC: Model-based classifier
NME: Non-mass-like enhancement
PACS: Picture archiving and communication system
ROC: Receiver operating characteristic
ROI: Region of interest
SDAC: Standard deviation of area covered by cancer-like voxels
SE-EPI: Spin-echo echo-planar imaging
VOI: Volume of interest
